# Supplementary material for: Splice-Junction-Based Mapping of Alternative Isoforms in the Human Proteome
Source: Cell Rep. Author manuscript; Available in PMC 2020 Jan 15. (PMC6961840; doi:10.1016/j.celrep.2019.11.026)
Supplement: 3 [file NIHMS1546469-supplement-3.zip › DF2/PXD0010154/Liver-45-P10636-AEEAGIGDTPSLEDEAAGHVTQAR.pdf]

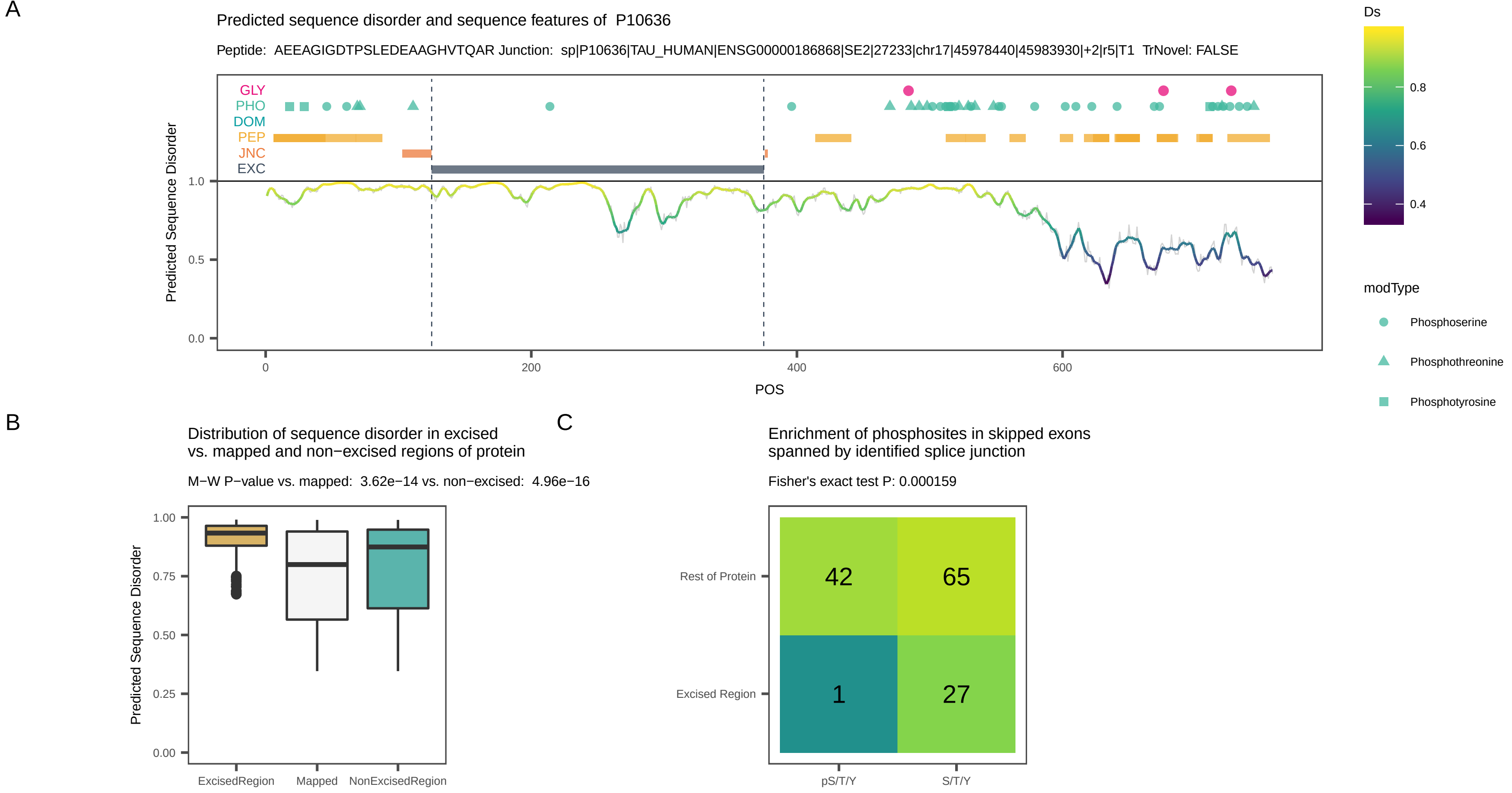

A
Predicted sequence disorder and sequence features of P10636
Peptide: AEEAGIGDTPSLEDEAAGHVTQAR Junction: sp|P10636|TAU_HUMAN|ENSG00000186868|SE2|27233|chr17|45978440|45983930|+2|r5|T1 TrNovel: FALSE
GLY
PHO
DOM
PEP
JNC
EXC
Predicted Sequence Disorder
1.0
0.5
0.0
0
200
400
600
POS
Ds
0.8
0.6
0.4
modType
Phosphoserine
Phosphothreonine
Phosphotyrosine
B
Distribution of sequence disorder in excised vs. mapped and non−excised regions of protein
M−W P−value vs. mapped: 3.62e−14 vs. non−excised: 4.96e−16
Predicted Sequence Disorder
1.00
0.75
0.50
0.25
0.00
ExcisedRegion
Mapped
NonExcisedRegion
C
Enrichment of phosphosites in skipped exons spanned by identified splice junction
Fisher's exact test P: 0.000159
Rest of Protein
Excised Region
42
65
1
27
pS/T/Y
S/T/Y
